# Evidence for the Gut Microbiota Short-Chain Fatty Acids as Key Pathophysiological Molecules Improving Diabetes

**DOI:** 10.1155/2014/162021

**Published:** 2014-08-17

**Authors:** Alessandra Puddu, Roberta Sanguineti, Fabrizio Montecucco, Giorgio Luciano Viviani

**Affiliations:** ^1^First Clinic of Internal Medicine, Department of Internal Medicine, University of Genoa, IRCCS Azienda Ospedaliera Universitaria San Martino, 6 Viale Benedetto XV, 16143 Genoa, Italy; ^2^Division of Cardiology, Foundation for Medical Researches, Department of Medical Specialties, Geneva University Hospitals, 64 Avenue de la Roseraie, 1211 Geneva, Switzerland; ^3^Division of Laboratory Medicine, Department of Genetics and Laboratory Medicine, Geneva University Hospitals, 4 Rue Gabrielle-Perret-Gentil, 1205 Geneva, Switzerland

## Abstract

In type 2 diabetes, hyperglycemia, insulin resistance, increased inflammation, and oxidative stress were shown to be associated with the progressive deterioration of beta-cell function and mass. Short-chain fatty acids (SCFAs) are organic fatty acids produced in the distal gut by bacterial fermentation of macrofibrous material that might improve type 2 diabetes features. Their main beneficial activities were identified in the decrease of serum levels of glucose, insulin resistance as well as inflammation, and increase in protective Glucagon-like peptide-1 (GLP-1) secretion. In this review, we updated evidence on the effects of SCFAs potentially improving metabolic control in type 2 diabetes.

## 1. Introduction

Type 2 diabetes (T2D) is a metabolic disorder characterized by hyperglycemia caused by the relative lack of insulin production, due to the exhaustion of pancreatic beta cell function after the establishment of insulin resistance [1]. The onset of inflammation in metabolic diseases (including obesity and diabetes) has been shown to be potentially related to alteration in the gut microbiota [2]. Since interindividual variations in the microbiome impact multiple human pathologies, understanding the factors that underlie changes in the composition of gut microbiota may be useful in designing more selective and effective therapies [3, 4]. Recently, a potential relationship between gut microbiota and T2D pathophysiology has been clearly suggested by two independent studies, which compared metagenomes from healthy and T2D subjects. For instance, an increase in Clostridium clostridioforme and a decrease in Roseburia_272 were demonstrated in T2D subjects from both Chinese and European populations [5, 6]. Accordingly, increased levels of Roseburia were associated with improved insulin sensitivity after gut microbiota transplantations from lean donors to recipients with metabolic syndrome [7]. These effects were potentially related to levels and activities of short-chain fatty acids (SCFAs). In particular, propionic and butyric acids were shown to reduce low-grade inflammation, to regulate cell proliferation and differentiation, and to induce hormone release [8–13]. Butyrate producing intestinal bacteria seems to play an important role in blood glucose regulation and lipid metabolism, as shown by fecal transplantation studies [7, 14]. Recently, Remely and coworkers demonstrated that distinct composition of gut microbiota producing different SCFAs may affect epigenetic gene regulation in obesity and T2D [15]. In addition, partial prevention of obesity through histone acetylation by SCFAs was reported by Duranton and colleagues more than ten years ago [16]. These beneficial effects of SCFAs were not only related to their property as histone deacetylase inhibitors, but also related to their activation of the transmembrane cognate G protein-coupled receptors [17].

## 2. Short-Chain Fatty Acids (SCFAs)

SCFAs are organic fatty acids with 1 to 6 carbon atoms existing in straight- and branched-chain conformations [18, 19]. SCFAs are produced in the distal gut by bacterial fermentation of macro-fibrous material that escapes digestion in the upper gastrointestinal tract and enters the colon, including resistant starch, dietary fiber, simple sugars, sugar alcohols, unabsorbed or undigested proteins, and endogenous substrates, such as sloughed off epithelial cells, mucus, intestinal enzymes, and other secretions [8, 20, 21]. Although common SCFAs include formic, acetic, propionic, butyric, isobutyric, valeric, isovaleric, and caproic acids [22], 90–95% of the SCFAs present in the colon are constituted by acetate, propionate, and butyrate [8], with intraluminal concentrations of about 60% acetate (C2), 25% propionate (C3), and 15% butyrate (C4) [23]. Butyrate is considered as a major energy source for the colonic epithelium. Indeed in the colon, butyrate oxidation occurs with an increased rate as compared to acetate and propionate [24]. Propionate, entering the portal circle, is primarily utilized in gluconeogenesis in the liver, whereas significant amount of acetate enters systemic circulation and reaches peripheral tissues. Consequently plasma levels of SCFAs are dominated by acetate [25, 26]. Absorption of SCFAs is rapid and the colon absorbs more than 95% of SCFAs [23], mainly through specific apical solute carriers such as the monocarboxylate transporter 1 (MCT1) and the sodium-coupled monocarboxylate transporter 1 (SMCT1) present in colonic epithelial cells [27]; consequently absorption of SCFAs contributes to maintain acid-base equilibrium and promotes the absorption of Na+. By providing a significant contribution to the total caloric intake, metabolized SCFAs concur to maintain energy homeostasis [28]. Interestingly, SCFAs of colon origin contribute approximately 5–10% towards human energy requirements [29]. Positive metabolic health effects (such as satiety increase, blood glucose, and cholesterol lower levels) have been shown after ingestion of resistant starch and have been associated with increased fecal SCFAs concentrations, particularly propionic and butyric acids [23, 30]. For instance, it has been shown that butyrate and propionate reduce food intake. In addition, butyrate plays important roles both at the intestinal level, by regulating transepithelial fluid transport, ameliorating mucosal inflammation and oxidative status, reinforcing the epithelial defense barrier, and preventing colorectal cancer, and at the extraintestinal level, by exerting ameliorating effects on many pathologies, including hemoglobinopathies, metabolic diseases, hypercholesterolemia, insulin resistance, and ischemic stroke [31].

Several studies demonstrated that differences in the rate and ratio of SCFA production depend primarily on the type of substrate [8, 32]. Ingestion of fermentable dietary fibers increased SCFA concentration, whereas the high-fat diet (HFD) reduced formation of SCFAs [23, 33]. In 2010, Freeland and colleagues observed that in hyperinsulinemic subjects receiving either a high-wheat fiber cereal or a low-fiber cereal daily for 1 year, concentrations of the acetate and butyrate were higher in the subjects on the high-fiber than the control cereal diet [34]. Furthermore, this effect was associated with improved metabolic control, suggesting the onset of protection from the deleterious effects of high fat diet-induced obesity and diabetes. Recently, the group of Jakobsdottir observed changed formation of SCFAs in rats administered with low-fat or high-fat diets supplemented with fermentable dietary fibers, showing that high-fat diet reduced the formation of butyrate, but increased succinate and cholesterol, as well as inflammation and liver fat in rats, whereas dietary fiber counteracts these effects [33]. In most of these studies the increased concentration of butyrate is associated with the improvement in metabolic health. This evidence was a start for many works investigating the potential clinical relevance for butyrate. Administration of butyrate in a rat model of insulin resistance and steatosis induced by HFD reduced liver steatosis and inflammation, normalized transaminases, insulin resistance, and glucose tolerance [35]. Interestingly treatment of diabetic rat with butyrate decreased plasma glucose, and HbA1c improved the beta-cell proliferation and plasma insulin levels [36].

## 3. SCFA Receptors

Besides acting as a local nutrient source, SCFAs can also trigger cell-specific signaling cascades by receptor activation. Cognate receptors for SCFAs were formally described through the process of “deorphanization” of known protein with unknown function. Receptors for SCFAs are two G-protein coupled receptors (GPCRs): FFAR2 (free fatty acid receptor2, previously known as GPR43) and FFAR3 (or GPR41) [37–40]. Although they share about 40% similarity [37, 38, 41], they differ in their specificity for ligands, with ligand potency affected by specie heterogeneity [37, 38], and for G protein coupling: FFAR3 couples exclusively through the pertussis toxin sensitive G_i/o_ family, whereas FFAR2 couples either the G_i/o_ and pertussis toxin-insensitive G_q_ families [38]. The intracellular signaling cascade triggers inositol 1,4,5-trisphosphate formation, intracellular [Ca^2+^] mobilization, activation of extracellular signal-regulated kinase 1/2, and inhibition of intracellular cAMP accumulation [38]. Both receptors are expressed in a variety of cells, including colonic enteroendocrine L cells [42–44], mucosal mast cells [43], adipose tissue [37, 38], neutrophils, and monocytes [45]. Activation of the receptors affects distinct function depending on their tissue distribution, for instance FFAR3 is involved in SCFAs-stimulated leptin production by adipocytes and lipid profiles regulation, whereas FFAR2 is involved in modulation of inflammation and Glucagon-like peptide-1 (GLP-1) secretion [36, 40, 46]. Kimura and coworkers demonstrated that FFAR2 is involved in controlling body energy utilization. Indeed FFAR2-deficient mice are obese on a normal diet, whereas mice overexpressing FFAR2 specifically in adipose tissue remain lean even when fed with a high-fat diet [47].

Interestingly, it has been recently reported that expression of FFARs may be affected by different compositions of gut microbiota through epigenetic regulation of gene expression [15]. In particular the promoter region of FFAR3 showed a significantly higher methylation in the lean control group compared to type 2 diabetics and to obese subjects. These interactions between microbiota and epigenetic regulation may cause changes in expression and signaling of FFARs and influence the onset of metabolic disease.

Deficiency of FFAR2 results in increasing or maintaining inflammation in models of colitis, arthritis, and asthma, related to increased production of inflammatory mediators and increased immune cell recruitment [48]. In FFAR2-deficient mice it has also been reported impaired GLP-1 secretion in responses to SCFAs and reduced basal levels of active GLP-1 when compared with controls [49]. Although deficiency of FFAR3 in mice has been reported to be associated with a lower expression of peptide YY (PYY) and an attenuated microbiota-induced secretion of PYY [50], FFAR3 seems to play a minor role in stimulation of GLP-1 secretion [49, 51] and is not required for butyrate- and propionate-dependent induction of Glucose-dependent insulinotropic peptide secretion from K cells [51].

## 4. SCFAs and GLP-1

GLP-1 is an incretin hormone that participates to glucose homeostasis, mainly by lowering plasma glucose concentration, improving insulin secretion and resistance, and preserving pancreatic beta-cell function [52, 53]. GLP-1 is secreted by the intestinal L cells, an open-type intestinal epithelial endocrine cells [54], in response to a variety of nutrients [55, 56].

SCFAs have been linked to increased GLP-1 secretion in both animal and human models [57–60]. The effect of SCFAs on GLP-1 release may be affected by different fiber feeding compositions and experimental setting [58, 61–63]. Moreover, Freeland and Wolever reported that in hyperinsulinemic subjects a long-term diet rich in SCFAs is needed to increase GLP-1 concentration. Interestingly rectal, but not intravenous, infusion of SCFAs was effective in acute increment of GLP-1 secretion [60]. Since GLP-1 secreting L cells are mainly located in the distal ileum and colon, and the primary site of SCFAs production is the colon, ability of SCFAs to induce GLP-1 secretion has been widely investigated. Expression of both FFAR2 and FFAR3 was reported in the colon, with particularly strong expression in GLP-1 producing L-cells [42–44, 64]. Evidence that SCFAs evoked the release of GLP-1 into plasma came more than 15 years ago from Dumoulin and coworkers [65]. However the intracellular mechanism linking SCFAs with GLP-1 release is not yet fully understood. Recently, Tolhurst and colleagues [49] demonstrated that in primary murine colonic cultures, expressing both mRNAs for FFAR2 and FFAR3, acetate and propionate stimulate GLP-1 secretion into the basolateral side through activation of FFAR2, via G_q/11_ and subsequent increased of the intracellular calcium level. Furthermore they found that FFAR2-deficient mice had significantly reduced GLP-1 protein content and reduced basal levels of active GLP-1, suggesting that FFAR2 may be involved in maintaining L-cell function.

Among SCFAs butyrate seems to have a slower potency than acetate and propionate in stimulating GLP-1 secretion [49]. However butyrate treatment of the human L-cell line NCI-H716 cells resulted in enhanced secretion of GLP-1 and increased expression of genes involved in GLP-1 synthesis and secretion [57]. Furthermore the beneficial effects of administration of the probiotic VSL#3 such as reduced food intake, protection from body weight gain and insulin resistance, and rise in GLP-1 secretion were associated with the increased levels of butyrate [57]. Mechanisms through which butyrate increases GLP-1 secretion are still matter of debate. The poor responsiveness to SCFAs of the GLP-1, secreting cell line GLUTag, has been associated with the very low expression of FFAR2 [49]. Conversely, Yadav and colleagues suggested that butyrate interaction on L-cells might be mediated via FFAR3, since rising in secretion of GLP-1 induced by butyrate was associated with increased expression of FFAR3 [57]. Moreover, butyrate-induced total GLP-1 secretion was attenuated in the FFAR3 knockout mice [51].

## 5. SCFAs and Insulin

Insulin is a peptide hormone produced by pancreatic beta cells, which regulates the level of blood glucose inducing cellular glucose uptake, especially by adipose and skeletal muscle cells, and inhibiting glycogen lysis in liver cells. Therefore, the balance between insulin secretion and insulin action maintains homeostasis of glucose. In some metabolic disorders, such as T2D, cells fail to respond to the normal actions of insulin, resulting in insulin resistance [1]. Evidence that SCFAs protect against diet-induced obesity and insulin resistance has been reported by many authors [51]. Changes in the microbiota, and consequently in SCFAs composition, have been hypothesized to be associated with the development of obesity, insulin resistance, and diabetes. Colonization of normal and germ-free mice with microbiota harvested from the caecum of obese mice results in increased body weight gain [66]. Oppositely, modulation of the gut microbiota composition leading to increased butyrate production results in suppression of body weight gain and insulin resistance in high fat diet-fed and obese mice [57]. Furthermore improved insulin sensitivity has been found after infusion of butyrate-producing intestinal microbiota from lean donors to male subjects with metabolic syndrome [7]. Interestingly activation of FFAR2 by SCFAs may suppress insulin signaling in adipocytes, inhibiting fat accumulation in adipose tissue, but promoting the metabolism of unincorporated lipids and glucose in other tissues [47].

Among SCFAs, butyrate seems to play an important role in the pathology of obesity and diabetes; therefore the attention of the most recently studies has been focused on it. Interestingly, consistent with pancreatic beta-cells stimulation by incretins, oral administration of sodium butyrate in mice significantly elevated plasma insulin [51]. Recently, a metagenomic study on obese versus lean subjects showed that butyrate-producing bacterial abundance was substantially decreased in obese subjects [6], supporting the fact that butyrate may be responsible of healthy metabolism. It has been widely recognized that a fiber enriched diet protects against obesity and insulin resistance. Supplementation of butyrate to the high-fat diet led to an increase in insulin sensitivity and a reduction in obesity in C57BL/6 mice [67]. Administration of the probiotic VSL#3 to high fat diet-fed (HFD) mice was associated with increased butyrate production from the microbiota, suppressed body weight gain and insulin resistance, and reduced fed blood glucose levels [57].

Regulation of insulin levels seems to depend on FFAR2 expression. Indeed lower insulin levels have been reported in FFAR2 knockout mice compared with wild-type controls after a prolonged period on a high-fat diet [68]. Furthermore reduced insulin levels and impaired glucose tolerance have been observed in the FFAR2 knockout mice during an oral glucose tolerance text [49]. Interestingly the reduced insulin levels were not associated with differences in insulin tolerance indicating that the observed impaired glucose tolerance may reflect an impairment of insulin secretion, probably due to the reduced circulating GLP-1 concentrations. However, finding that FFAR2 is expressed in pancreatic beta-cells [69] suggested that FFAR2 may be involved in direct regulation of SCFAs on islet cell function. This hypothesis is supported by certain studies about effect of butyrate on pancreatic beta-cell differentiation and function. In 2006, Goicoa and coworkers reported that short exposure of embryonic stem cells to sodium butyrate activates early pancreatic development genes and increases the beta-cell differentiation [70]. Furthermore it has been reported that nestin-EGFP-positive progenitor cells (NPPCs) cultured in the presence of GLP-1 and sodium butyrate increased levels of transcripts encoding pancreatic developmental factors and insulin, leading to differentiation of NPPCs into insulin-producing cells [71]. These promising results may be related to the activity of sodium butyrate as histone deacetylases (HDAC) inhibitor; indeed it is well known that HDAC inhibitors promote beta-cell development, proliferation, differentiation, and function [72]. Furthermore, in a rat model of juvenile diabetic treatment with sodium butyrate improved glucose homeostasis as well as beta-cell proliferation and function and reduced beta-cell apoptosis [36].

## 6. SCFA and Inflammation Associated with Diabetes

TDM2 is characterized by low-grade inflammation with increased levels of cytokines, such as interleukin (IL)-6, IL-1, or tumor necrosis factor-alpha (TNF-*α*). These inflammatory molecules are upregulated in insulin-target tissues, including the liver, adipose tissue, and muscles [73], thus contributing to insulin resistance [74, 75].

It has been reported that alterations in the intestinal microbiota composition promote a proinflammatory state of the adipose tissue that is associated with obesity and subsequent insulin resistance [76]. The composition of the gut microbiota, and consequently of its metabolic products, is mainly affected by dietary changes [77–79]; an appropriate intake of dietary fibers is often associated with a SCFA profile that might increase anti-inflammatory response in the body, whereas a high-fat diet was shown to be associated with reduction of SCFAs and increase in lipopolysaccharide (LPS) levels [80], suggesting a colonization of Gram-negative bacteria. LPS was shown to induce the release of proinflammatory molecules [81], which contributed to the establishment of increased permeability and inflammation in the intestinal epithelium. This condition is known as “metabolic endotoxemia.” On the contrary, incubation of a human colonic epithelial cell line with butyrate was shown to increase transepithelial resistance by promoting the assembly of tight junction [82, 83]. The compromised epithelial integrity allows movement of bacteria and/or dissemination of their products from the gut lumen to tissues, thus resulting in the increase of systemic inflammation. Therefore, LPS released by gut microbiota may pathophysiologically affect the function of other organs, further increasing insulin resistance. LPS receptors were also identified to mediate critical activities potentially underlying insulin resistance. For instance, activation of Toll-like receptor 4 (TLR4) in pancreatic islets was shown to increase proinflammatory cytokine production (both in activated macrophages and in beta-cells) that impaired function and decreased viability of beta-cells [84–86]. Indeed, TLR4 activation directly resulted in a decreased mRNA expression of pancreas-duodenum homebox-1 (PDX-1), insulin gene expression, reduced insulin content, and diminished insulin-induced glucose secretion. In addition, LPS upregulated the expression of nuclear factor kappa-light-chain-enhancer of activated B cells (NF-*κ*B) and activated mitogen-activated protein kinase (MAPK)-mediated pathways in adipocytes [87]. The upregulation of TLR4 mRNA levels induced by HFD in the liver was shown to be counteracted by butyrate [35]. High-fat diet was associated with the upregulation of TNF-*α* and phosphorylation of NF-*κ*B in the ileum [88], confirming that fat intake might increase mediators of intestinal permeability and inflammation. The dietary fibers can partly counteract these harmful effects, probably through production of SCFAs, particularly propionic and butyric acids, that could have anti-inflammatory effects in the body [33, 89]. The anti-inflammatory effects of SCFAs are probably due to a balance between suppression of proinflammatory mediators, such as TNF-*α* and IL-6, and induction of anti-inflammatory cytokines. For instance, administration of butyrate to HFD fed animals significantly reduced hepatic expression of TNF-*α*, IL-1*β*, and IL-6, therefore reducing liver steatosis and inflammation [35]. On the other hand, butyrate was also shown to abrogate secretion of the proinflammatory cytokines IL-12 and TNF-*α* and increase the release of the anti-inflammatory cytokine IL-10 by monocytes exposed to bacteria [90]. Finally, propionate and butyrate reduced expression of the proinflammatory cytokines TNF-*α* and IL-6 in human adipose tissue [89]. The ability of SCFAs to reduce low-grade inflammation is related to their capacity in modulating leukocyte and adipocyte function, thus reducing expression and production of inflammatory cytokines and chemokines [35, 48, 89, 91, 92]. Tedelind demonstrated that SCFAs decreased neutrophil release of TNF-*α* induced by LPS [93]. Accordingly, the exposure of adipocytes to propionic acid significantly downregulated several inflammatory cytokines and chemokines (such as TNF-*α*, resistin, and CCL5) [13, 94].

The molecular mechanisms through which SCFAs exert their anti-inflammatory effects may be related to the activation of their cognate G protein-coupled receptors FFAR2 and FFAR3, in addition to their function as HDAC inhibitors [95]. It has been reported that SCFAs might affect leukocyte subsets, including polymorphonuclear (PMN) cell activation [96–98]. FFAR2 seems to be a critical molecule regulating these effects. Firstly, FFAR2 mRNA is predominantly expressed in immune cells, particularly in PMN cells [37–39]. Moreover it has been reported that FFAR2 is induced during the differentiation of leukocyte progenitor cells to monocytes or neutrophils, suggesting that it could have an important function in the differentiation and/or activation of leukocytes [45]. Furthermore, Maslowski and colleagues demonstrated that inflammatory responses are regulated by interaction between SCFAs and FFAR2 [48], showing that immune cells from FFAR2-deficient mice have increased production of inflammatory mediators in comparison with wild-type mice. SCFA-mediated activation of FFAR2 was shown to trigger recruitment of circulating leukocytes to the inflammatory site via activation of intracellular signaling pathways including MAPK, Protein Kinase C (PKC), and Phospholipase C (PLC) [95]. On the other hand, by acting as HDAC inhibitors, SCFAs modulated the transcription of several target genes leading to downregulation of the expression of CXC chemokine receptor 2 (CXCR2) and inhibiting migration of neutrophils [48]. Finally, butyrate was shown to inhibit macrophage migration in response to LPS [99].

Another anti-inflammatory mechanism mediated by SCFAs was represented by adhesion molecule downregulation on endothelial cells, finally resulting in inhibition of leukocyte migration to inflammatory sites [78, 82]. By preventing inflammatory cell adhesion and chemotaxis, SCFAs were able to reduce immune cell infiltration to adipose tissue [100]. In addition, butyrate was shown to reduce TNF-*α*-induced VCAM-1 expression on umbilical vascular endothelial cells (HUVECs). Accordingly, preincubation with propionate and butyrate reduced both surface expression and mRNA levels of VCAM-1 in TNF-*α*- and IL-1*β*-stimulated HUVECs in a dose-dependent manner [100].

## 7. Conclusion

**Figure 1:**
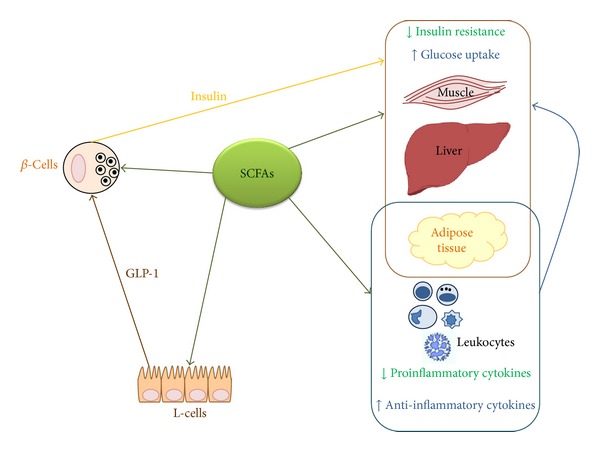
SCFAs improve metabolic functions in T2D. SCFAs were shown to affect pancreatic beta-cell function by directly acting as HDAC inhibitors (promoting *β*-cell development, proliferation, and differentiation) or by indirectly increasing GLP-1 secretion from enteroendocrine L-cells (leading to insulin release). Furthermore, SCFAs reduce the release of proinflammatory cytokines by adipose tissue and weaken leukocyte activation. These anti-inflammatory effects improve insulin resistance, tissue glucose uptake, and blood glucose levels.

SCFAs have been associated with improvement of metabolic functions in T2D (including the control of blood glucose levels, insulin resistance, and GLP-1 secretion). These effects result from to the different tissues expressing SCFA receptors and, thus, are capable of responding to the beneficial effects induced by these molecules. Evidence reviewed in our paper indicates that regulation of blood glucose concentrations may involve several positive effects exerted by SCFAs occurring at different levels (Figure 1): (i) the decreased inflammatory state that reduces insulin resistance, (ii) the contemporarily increased GLP-1 secretion that stimulates insulin release, and (iii) the improved beta-cell function that contribute to amelioration of glucose homeostasis.
